# The Enigma of the Dichotomic Pressure Response of GluN1-4a/b Splice Variants of NMDA Receptor: Experimental and Statistical Analyses

**DOI:** 10.3389/fnmol.2016.00040

**Published:** 2016-06-10

**Authors:** Alice Bliznyuk, Gideon Gradwohl, Michael Hollmann, Yoram Grossman

**Affiliations:** ^1^Department of Physiology and Cell Biology, Zlotowski Center for Neuroscience, Ben-Gurion University of the NegevBeer-sheva, Israel; ^2^Department of Physics, Jerusalem College of TechnologyJerusalem, Israel; ^3^Department of Biochemistry I-Receptor Biochemistry, Ruhr University BochumBochum, Germany

**Keywords:** HPNS, CNS hyperexcitability, pressure, diving physiology, oocyte

## Abstract

Professional deep-water divers, exposed to hyperbaric pressure (HP) above 1.1 MPa, develop High Pressure Neurological Syndrome (HPNS), which is associated with central nervous system (CNS) hyperexcitability. It was previously reported that HP augments *N*-methyl-D-aspartate receptor (NMDAR) synaptic response, increases neuronal excitability and potentially causes irreversible neuronal damage. Our laboratory has reported differential current responses under HP conditions in NMDAR subtypes that contain either GluN1-1a or GluN1-1b splice variants co-expressed in *Xenopus laevis* oocytes with all four GluN2 subunits. Recently, we reported that the increase in ionic currents measured under HP conditions is also dependent on which of the eight splice variants of GluN1 is co-expressed with the GluN2 subunit. We now report that the NMDAR subtype that contains GluN1-4a/b splice variants exhibited “dichotomic” (either increased or decreased) responses at HP. The distribution of the results is not normal thus analysis of variance (ANOVA) test and clustering analysis were employed for statistical verification of the grouping. Furthermore, the calculated constants of alpha function distribution analysis for the two groups were similar, suggesting that the mechanism underlying the switch between an increase or a decrease of the current at HP is a single process, the nature of which is still unknown. This dichotomic response of the GluN1-4a/b splice variant may be used as a model for studying reduced response in NMDAR at HP. Successful reversal of other NMDAR subtypes response (i.e., current reduction) may allow the elimination of the reversible malfunctioning short term effects (HPNS), or even deleterious long term effects induced by increased NMDAR function during HP exposure.

## Introduction

Diving to depths of more than 50 m of seawater (msw), (0.1 MPa = 1 ATA ≅ 10 msw) is considered as deep diving and is mainly used for occupational and military purposes. Professional divers in the oil industry perform dives to depth of 200 msw (2.1 MPa) or even deeper in order to maintain and recover oil platform equipment. The diving depth record for commercial diving was achieved in 1988 in the Mediterranean Sea when professional divers performed a pipe line connection at a depth of 534 msw (5.37 MPa; Ciesielski and Imbert, 1989). The deepest test dive was made in a dry pressure chamber to a depth of 701 msw (7.05 MPa) by the company Comex in 1992 (Lafay et al., 1995).

Under such condition professional divers may suffer from direct hyperbaric pressure (HP) effects that present many physiological challenges, affecting the lungs, viscera, and nervous system. Hydrostatic HP above 1.1 MPa causes High Pressure Neurological Syndrome (HPNS; Bennett and Rostain, 2003), which is characterized by reversible central nervous system (CNS) hyperexcitability and cognitive and motor deficits. HPNS susceptibility and symptoms intensity depend on the compression rate and on the absolute ambient pressure. It is conceivable that this constellation of signs and symptoms origins in disturbances in synaptic activity of neuronal networks (Grossman et al., 2010). In addition to HPNS, prolonged repetitive deep sea operations of professional divers at HP may result in permanent memory and motor impairment (Gronning and Aarli, 2011).

Earlier pharmacological studies have repeatedly implicated the *N*-methyl-D-aspartate receptor (NMDAR) in CNS hyperexcitability as part of HPNS (Fagni et al., 1985, 1987; Zinebi et al., 1988, 1990; Daniels and Grossman, 2003; Mor and Grossman, 2010). In recent years a large body of data has been accumulated on NMDAR structure and function.

NMDARs belong to the family of ionotropic glutamate receptors that mediate excitatory neuronal transmission within the CNS (Traynelis et al., 2010). There are 14 different subunits of NMDARs: the eight GluN1-1a to -4a and GluN1-1b to -4b subunits result from alternative RNA splicing (Dingledine et al., 1999). The four GluN2A to GluN2D subunits are encoded by four different genes, while the two GluN3A and GluN3B subunits are encoded by two genes. Conventional NMDARs are assembled from different combinations of GluN1 and GluN2 subunits in a heterotetrameric “dimer of dimers” structure (Furukawa et al., 2005; Paoletti, 2011). Different NMDAR subtypes have specific spatiotemporal distribution and function(s) in the CNS (Watanabe et al., 1992; Akazawa et al., 1994; Laurie and Seeburg, 1994; Monyer et al., 1994; Sheng et al., 1994; Takai et al., 2003; Paoletti, 2011). Studies on recombinant, heterologously expressed NMDARs, have revealed how the subunit composition endows each subtype with unique biophysical and pharmacological properties (Paoletti et al., 2013; Sanz-Clemente et al., 2013).

Electrophysiological studies in rat brain slices at HP, preceded by the pharmacological studies, showed a significant increase in the synaptic NMDAR response followed by postsynaptic excitability changes (Mor and Grossman, 2006, 2007) and reduced efficiency of Mg^2+^ blockade (Mor and Grossman, 2007). Lately, molecular studies from our laboratory (Mor et al., 2012) have revealed differential current responses under HP conditions in NMDAR subtypes that contain either GluN1-1a or GluN1-1b splice variants co-expressed in frog oocytes, with all four GluN2 subunits. Recently, we also reported (Bliznyuk et al., 2015) that the HP-dependent increase in ionic currents is also dependent on the specific splice variant of GluN1 co-expressed with GluN2A subunit. In addition, we observed that receptors containing GluN1-4a or GluN1-4b splice variants mediate opposite HP responses, an increase or a decrease of the current (Bliznyuk et al., 2015). However, in only 30% of the experiments the current was decreased at HP.

The goal of the present study is to investigate the opposing responses of GluN1-4 in an attempt to understand their possible underlying mechanism(s), such as over-expression, variable stoichiometry, or a GluN2-dependent mechanism and to verify that it is not a random distribution of responses. Revealing the mechanism of such decreased response is of great value since the contribution of NMDARs to HPNS is primarily due to the increase of their activity at HP. Therefore, the possibility of turning the receptors into the decreased response mode, or just increasing their frequency in the “normal” population of other GluN1 subunits, may prove efficient in reducing or eliminating HPNS symptoms.

## Materials and Methods

Most of the methods have been described in our recent publication (Bliznyuk et al., 2015). A summary of essential methodology principles is repeated below, for reading convenience.

### Oocyte Preparation

Stage V and VI oocytes were surgically removed from *Xenopus laevis* ovaries (anesthetized with 1.5 g/L ethyl 3-aminobenzoate methanesulfonate salt; Sigma-Aldrich, Israel), prepared and maintained in ND-96 solution (at 18°C) containing (in mM): 96 NaCl, 2 KCl, 1 MgCl_2_, 1.8 CaCl_2_, 2.5 sodium pyruvate, 5 HEPES, 10 mg/ml PEN/STREP, and 50 μg/ml gentamicin adjusted to pH 7.5. The incisions were closed by absorbable sutures and the animals were returned to the tank. Surgery was performed according to the guidelines laid down by the Ben-Gurion University of the Negev ethics committee for the care and use of animals for experimental work (IL-69-12-2011). Within 24 h after surgery, oocytes were injected with one of the two newly synthesized GluN1-4a/b splice variant cRNAs (5 or 0.2 ng) and one of the GluN2A/B subunit cRNA (5 or 0.2 ng, see text for the specific experiments) using a nanoliter injector (World Precision Instruments, Sarasota, FL, USA). All cRNAs were produced by Prof. M. Hollmann’s laboratory (Ruhr University, Bochum, Germany). The NMDAR cDNA accession numbers are: GluN1-4a: U08267; GluN1-4b: U08268; GluN2A: AF001423 and GluN2B: U11419. All NMDAR subtypes were successfully expressed on the oocytes’ membranes. After incubation for 3–4 days, individual oocytes were placed in a custom-designed recording bath, inserted into a pressure chamber, and superfused at constant velocity (7–8 ml/min) with frog physiological solution containing (in mM): 90 NaCl, 1 KCl, 1.5 BaCl_2_, 10 HEPES, and zero added Mg^2+^ in order to remove the known physiological Mg^2+^ blockade of NMDARs. The solutions were introduced into the pressure chamber by means of a high-pressure pump (“minipump”, LDC Analytical Inc., Riviera Beach, FL, USA). Each NMDAR subtype testing included at least two separate batches of oocytes obtained from different frogs.

### Pressure, Compression, and Decompression

The pressure chamber, perfusion system, helium compression, and the experimental setup were described in detail in Mor and Grossman (2006) and recently by Bliznyuk et al. (2015). Briefly, experiments were carried out in a pressure chamber (Canty Assoc., NY, USA). HP was attained by compressed helium, a gas that is chemically inert at our experimental pressure range of 0.1–5.1 MPa. Rates of compression/decompression varied between 0.1 and 1.0 MPa/min. To avoid transient effects of pressure (Grossman and Kendig, 1984), recordings were taken at strictly controlled ambient temperature (25 ± 1°C). The typical time to reach 5.1 MPa and stable temperature conditions was 20–25 min, including the time needed for stabilization of temperature transients of ±1–3°C during compression and decompression. This pressure step is used routinely in our laboratory to consistently demonstrate HP effects. Recovery at 0.1 MPa was always attempted. Oocytes that didn’t survive decompression were excluded from the data pool.

### NMDAR Current Recordings

Oocytes were voltage-clamped at −70 mV employing the two-sharp electrode voltage clamp technique, using GeneClamp 500- amplifier (Molecular Devices, Axon Instruments Inc., CA, USA). The co-agonists glutamate (100 μM, Sigma, Israel) and glycine (10 μM, Sigma, Israel) were added to the physiological solution and applied for a 20 s exposure for measuring the Ba^2+^ currents through the receptors (Bliznyuk et al., 2015). Leak (baseline) currents were subject to change during the compression and decompression procedures, and they sometimes differ under hyperbaric vs. control (0.1 MPa) conditions. Nonetheless, under constant conditions (pressure, temperature, solution flow rate, pH, etc.), leak currents are stable and thus they can be subtracted from NMDAR responses. Oocytes membrane holding potential (−70 mV) was monitored continuously; deviations of up to ±1 mV were accepted. Time control protocols for 2–3 h were carried out and showed oocytes stability under control, HP, and decompression conditions in our previous studies (Mor et al., 2012; Aviner et al., 2014; Bliznyuk et al., 2015). As noted above, currents were acquired under control (0.1 MPa) and hyperbaric (5.1 MPa) conditions and analyzed offline.

In order to study the possibility that the receptor conductance changes under HP conditions, I/V curves were recorded (depicting the input conductance of the oocytes). This was achieved by applying 2 s voltage ramps from −150 to +50 mV (via the clamp amplifier) during maximal steady-state currents. The same voltage ramps were recorded during the closed state of the receptor, without applying the co-agonists glutamate and glycine to the solution, so they could be subtracted from the maximal recording during offline analysis.

In order to study whether a change in glutamate affinity at HP is involved in the current modulation, dose response curves were constructed. Glutamate concentration varied from 0 μM to 5, 30, and 100 μM, while the glycine concentration remained unchanged at 10 μM.

### Statistical Analysis

In each experiment, control and HP conditions were applied to the same oocyte. In other words, the same oocyte was exposed to the same pH, temperature, solution concentrations, flow rate, and agonist concentration conditions. The only difference was the HP exposure value. At each pressure step, the same agonist application was repeated a few times. At least 2–3 very similar current traces were acquired under the same conditions and were averaged. The results of maximal steady-state current amplitude measurements are expressed as mean amplitude ± SEM (standard error of mean); *n* denotes the number of experiments (number of oocytes) for each experimental protocol. The degree of significance is denoted by the value of *p*, and is considered statistically different when *p* < 0.05. As mentioned, each NMDAR subtype was tested using at least two separate batches of oocytes obtained from different frogs. Each batch of oocytes of any subtype of NMDAR exhibited two opposing responses at HP an increase or a decrease of the current. A χ^2^ test was applied where appropriate in order to reject/accept the hypothesis of random (50–50%) distribution of these negative and positive responses. If random response was rejected, we used “*k*-means cluster analysis” (Gan et al., 2007) that divided the data in clusters of responses and validated the different populations by the one-way analysis of variance (ANOVA) test with OriginLab 8 software function “K means cluster analysis” (OriginLab Corp., Northampton, MA, USA) and MatLab^®^ function “*k*-means.m” (see below).

Graphical representations were made using OriginLab 8 software or MatLab^®^.

Dose response curve fit calculations were made using OriginLab 8 software function “Growth/Sigmoidal/DoseResp”.

### Matlab Analysis

#### The Kolmogorov-Smirnov Test

MatLab^®^ “KStest.m” (Chakravarti et al., 1967) was used to decide whether sample *X1*,…,*Xn* belongs to a normally distributed population. It is accomplished by comparison of the observed cumulative distribution functions (CDF) to the expected normal CDF. After applying MatLab^®^ KStest, if *p* < 0.05, the given distribution is said to be normal.

#### Clustering Analysis

MatLab^®^ function “*k*-means.m” was also used for clustering analysis (Hartigan and Wong, 1979; Theodoridis and Koutroumbas, 2008). This method partitions *n* observations into *k* clusters (*k* is determined by the user), in which each observation belongs to the cluster with the nearest distance to the cluster’s centroid. In the second step, the silhouette values are calculated in order to determine how well each observation fits the corresponding cluster. The silhouette value for each point is a measure of how similar that point is to points in its own cluster, when compared to points in other clusters. The silhouette value for the i’th point, *S*_i_, is defined as: *S*_i_ = (*b*_i_ − *a*_i_)/max(*a*_i_, *b*_i_) where *a*_i_ is the average distance from the i’th point to the other points in the same cluster as i, and *b*_i_ is the minimum average distance from the ith point to points in a different cluster, minimized over clusters.

The silhouette value ranges from −1 to +1. A high silhouette value indicates that i is well-matched to its own cluster, and poorly-matched to neighboring clusters. If most points have a high silhouette value, then the clustering solution is appropriate. If many points have a low or negative silhouette value, then the clustering solution may have either too many or too few clusters. The silhouette clustering evaluation criterion can be used with any distance metric.

#### α-Stable Distribution

MatLab^®^ function “stblfit.m” was used in an attempt to describe an alternative distribution for our results. The α-stable is a four-parameter family of distributions and is denoted by *S*(α, β, γ, δ). A characteristic function provides a description of a function of a random variable. In general, α denotes the tail of the distribution, β denotes the skewness, γ denotes the scale, and δ denotes the location. Mathematically, the characteristic function Φ of *S* is defined by:

(1) $$\Phi (t)=\text{exp}\left(-\gamma^{\alpha }{\left|t\right|}^{\alpha }\left(1-i\beta \;*\;\text{sign}(t)\tan\left({}^{\pi \alpha }\text{​}/2\right)\right)+i\delta t\right)$$

(When *t* is the argument of the characteristic function; the sign is 1 for positive numbers, −1 for negative numbers and 0 for 0).

## Results

### Current (I) Analysis

#### New Approach for Analysis

**Figure 1:**
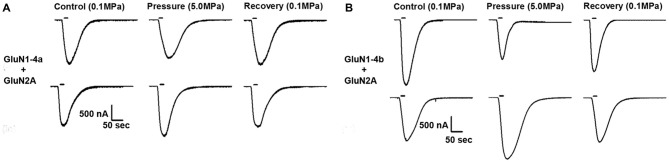
**Different hyperbaric pressure (HP) effects on GluN1-4a/b splice variants. GluN1-4a (A) and GluN1-4b (B) splice variants were co-expressed with the GluN2A subunit.** Currents for each of the splice variants were either augmented (lower traces) or decreased (upper traces) at HP. For all experiments the applied co-agonists were glutamate (100 μM) and glycine (10 μM) with no [Mg^2+^]_o_ added. The 20 s agonists application time is indicated by horizontal bars. The HP effects are reversed after full decompression for all subtypes.

Reexamination of our previously published data of GluN1-4a/b+GluN2A subtype responses to HP exposure (Bliznyuk et al., 2015) with the χ^2^ test, while *assuming* that the opposing responses belong to two different groups (see examples in Figure 1, reveals that it is not coincidental. We reject the hypothesis of random (50–50%) distribution of these negative and positive responses. Using the Kolmogorov-Smirnov test (MatLab^®^ function “*k*-means.m”) we found that our data do not distribute normally. The presence of “positive” and “negative” responses, and the verification with the χ^2^ test, suggested the possibility of different clusters of responses. In order to justify this assumption mathematically, we performed clustering analysis using MatLab^®^ “*k*-means.m” function (see “Materials and Methods” Section; on the data presented in Table 1; experiments that showed a response bigger than 2SD, were not included). Initially, the algorithm performed analysis on an estimated number of two groups (*k* = 2 clusters). The silhouette values of positive and negative groups were very satisfactory. Almost all points received silhouette values higher than 0.5.

**Table 1:** **Quantitative analysis of the GluN1-4a/b+GluN2A currents at HP**.

Subunit composition	Amplitude at 0.1 MPa (nA) Mean ± SEM	Amplitude at 5.0 MPa (nA) Mean ± SEM	Amplitude (%Δ) Mean ± SEM	*n*	*p*
GluN1-4a + GluN2A	1103.50 ± 117.42	1383.68 ± 157.41	26.36 ± 4.49	12	<0.001
	**1105.90 ± 91.96**	**835.73 ± 150.53**	**−25.81 ± 10.12**	**5**
GluN1-4b + GluN2A	2011.25 ± 243.28	2784.48 ± 344.84	39.35 ± 5.62	13	<0.001
	**2797.33 ± 654.90**	**1648.41 ± 463.87**	**−43.42 ± 8.07**	**4**

*Current amplitudes are expressed as mean ± SEM under control (0.1 MPa) and HP conditions (5.1 MPa), mean %Δ change ± SEM of amplitude; n, number of experiments; p, degree of statistical significance for ANOVA-one way test, which tested the division of the results in two clusters of responses (an increase or a decrease of the current at HP). Both subtypes of the receptor exhibited two opposing response types, showing either an increase (normal font) or a decrease (bold font) of the current at HP. Each NMDAR subtype was tested with at least two separate batches of oocytes obtained from different frogs, while each batch showed the two types of response*.

The GluN1-4a variant showed an increase in 12 of 17 tested oocytes (+26.36 ± 4.49%), while 5 of 17 showed a decrease (−25.81 ± 10.13%). Similar responses were observed for the GluN1-4b variant: 13 of 17 oocytes showed an increase (53.62 ± 10.05%) while 4 of 17 oocytes exhibited current reduction (−43.42 ± 8.07%). Applying the ANOVA-one way test in order to verify that the two groups came from “two different populations” (using “K means cluster analysis” function of OriginLab software) we validated the different populations. Both variants showed *p*-values <0.001 (Table 1).

#### Type of GluN2 Subunit

In order to determine whether the phenomenon is dependent on a specific GluN2 subunit, we co-expressed GluN1-4a with GluN2B (5 ng of each subunit). Once again opposing HP responses were observed (Table 2): four of eight tested oocytes showed increased current (+8.07 ± 2.76%) while four of the eight oocytes exhibited decreased current (−22.67 ± 8.72). Although the ratio between “increase” and “decrease” groups and the current change at HP differ from the GluN1-4a with GluN2A subtype, it is conceivable that GluN2 subunits are not responsible for the opposing response *per se*. Using the same methods of clustering analysis (as in “New Approach for Analysis” Section) we obtained *p* = 0.001 that strongly suggest existence of two groups of responses.

**Table 2:** **Quantitative analysis of the currents at HP under various expression conditions**.

Subunit composition	Amplitude at 0.1 MPa (nA) Mean ± SEM	Amplitude at 5.0 MPa (nA) Mean ± SEM	Amplitude (%Δ) Mean ± SEM	*n*	*p*
**5 ng of each subunit**
GluN1-4a+GluN2B	1551.46 ± 273.63	1672.46 ± 290.87	8.07 ± 2.76	4	0.001
	**988.47 ± 207.86**	**845.94 ± 172.61**	**−14.29 ± 3.11**	**4**
**0.2 ng of each subunit**
GluN1-4a+GluN2A	225.63 ± 89.49	329.50 ± 112.85	57.19 ± 12.68	4	0.003
	**569.02 ± 121.91**	**222.76 ± 115.12**	**−59.54 ± 18.84**	**3**
GluN1-4b+GluN2A	285.37 ± 79.59	390.35 ± 138.27	30.88 ± 18.09	3	0.003
	**322.30 ± 92.74**	**140.53 ± 35.80**	**−54.31 ± 4.75**	**4**
**Different amounts of each subunit**
GluN1-4a (0.2 ng) + GluN2B (5 ng)	291.45 ± 165.73	446.55 ± 244.22	57.26 ± 10.55	5	0.005
	**314.90 ± 175.42**	**244.96 ± 159.68**	**−28.16 ± 10.69**	**2**
GluN1-4a (5 ng) + GluN2B (0.2 ng)	66.95 ± 4.65	85.84 ± 3.96	29.49 ± 7.87	4	<0.001
	**139.93 ± 22.09**	**90.53 ± 21.21**	**−37.13 ± 6.12**	**6**

*Current amplitudes are expressed as mean ± SEM under control (0.1 MPa) and HP conditions (5.1 MPa), mean %**Δ** change ± SEM of amplitude; n, number of experiments; p, degree of statistical significance for ANOVA-one way test, which tested the division of the results in two clusters of responses (an increase or a decrease of the current at HP). All variations of the expression (amount of cRNA injection and subunit combinations) exhibited two opposing response types, showing either an increase (normal font) or a decrease (bold font) of the current at HP. Each NMDAR subtype was tested with at least two separate batches of oocytes obtained from different frogs, while each batch showed the two types of responses*.

#### Level of Expression

Routinely, we injected 5 ng of each subunit per oocyte. This may have caused an over expression of the receptors that could lead to cluster formation on the oocyte’s membrane. In order to prevent possible “receptor clustering”, as a first approximation we reduced the amount of injected cRNA to 1 ng of each subunit. This did not reduce the observed total current (data not shown). We further reduced the amount of injected cRNA of both subunits to 0.2 ng. In this case the total current was greatly reduced, but the phenomenon of opposing HP responses (Table 2) was still observed. GluN1-4a splice variant showed current increase in four of seven tested oocytes (57.19 ± 12.68%), while the remaining three of the seven oocytes showed a decrease (−59.54 ± 18.84%). The *p*-value of 0.003 strongly suggested existence of two groups of responses. Similar responses were observed for the GluN1-4b variant: three of seven tested oocytes showed current increase (+30.88 ± 18.09%) while the remaining four of the seven oocytes showed a decrease (−54.31 ± 4.75%, *p* = 0.003). There was no significant difference between the two splice variants in this respect. These data, taken together with the rest of the responses, suggest that “receptor clustering” cannot explain the “opposing response”.

#### Variable Stoichiometry

It has been estimated that NMDARs are assembled from different combinations of two GluN1 and two GluN2 subunits in a heterotetrameric “dimer of dimers” structure (Furukawa et al., 2005; Paoletti, 2011). This does not completely exclude the possibility that different stoichiometries may randomly occur. In order to test whether a variable subunit-stoichiometry can explain the opposing HP response, different concentrations of each subunit were co-expressed (0.2 ng of GluN1-4a co-expressed with 5 ng of GluN2B and vice versa, Table 2). This resulted in small total currents, similar to the expression of 0.2 ng of both subunits (see above). Yet, the opposing response was still observed. Injection of 0.2 ng of GluN1-4a with 5 ng of GluN2B showed current increase in five of seven oocytes (+57.26 ± 10.55%) and decreased currents in remaining two of the seven oocytes (−28.16 ± 10.69%). The invers expression ratio of 5 ng of GluN1-4a with 0.2 ng of GluN2B showed current increase in 4 of 10 oocytes (+29.49 ± 7.87%) and decreased currents in the remaining 6 of the 10 oocytes (−37.13 ± 6.12%). These experiments suggest that variable subunit-stoichiometry of the receptors cannot explain the “opposing response”.

#### Control Experiments

It is important to note that in every batch of oocytes control oocytes were injected with only GluN1 splice variant (5 ng). We have never observed any measurable currents at our levels of sensitivity (data not shown). These controls were taken in order to insure that there is no interference of endogenous *Xen*NR2 in our experiments, which is known to be expressed in oocytes at mRNA level (Terhag et al., 2010).

**Figure 2:**
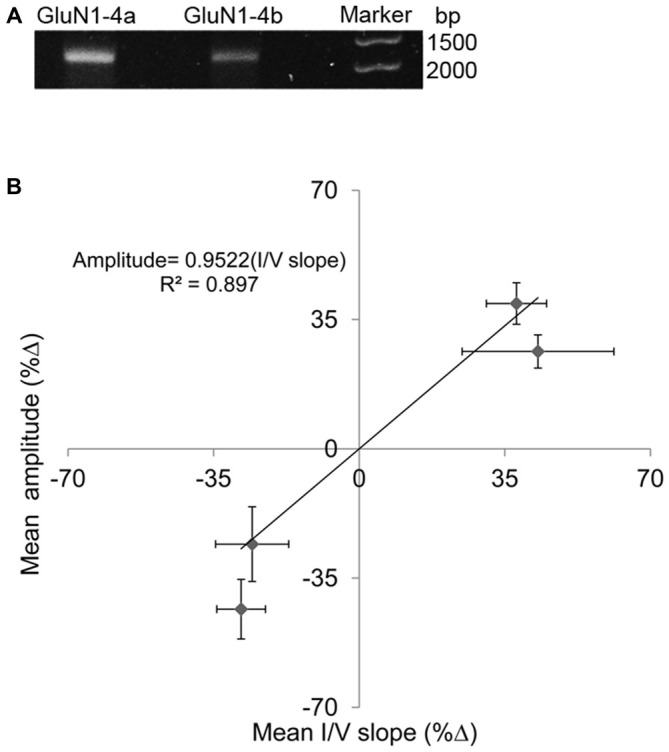
**(A)** cRNA-quality check. Single bands indicate stable cRNA (no fragmentation) **(B)** Correlations between amplitude and I/V slope changes at HP. Percent changes (%Δ) between HP and controls. Each point represents one subunit combination (either GluN1-4a or -4b co-expressed with GluN2A) with its SEM for amplitude change and corresponding I/V slope change that exhibited increased (2 points) or corresponding decreased (2 points) response. The slope of about 0.95 suggests that the change in input conductance is sufficient to explain the current amplitude change.

As a matter of precaution, we tested our cRNA stability. We found no damage or fragmentation in our samples (Figure 2A). Thus, cRNA modification cannot explain the “opposing response”.

### Input Conductance Analysis

We have previously shown (Bliznyuk et al., 2015) that the oocytes input conductance increases under HP conditions. However, further studies of the splice variants GluN1-4a and GluN1-4b co-expressed with GluN2A revealed two opposing results (Table 3): we observed an increase in input conductance in 9 of 14 measurements (42.97 ± 18.24%) and a decrease in the remaining 5 of the 14 oocytes (−25.73 ± 8.79%) in the GluN1-4a variant. In the GluN1-4b variant we observed an increase in 7 of 11 oocytes (37.81 ± 7.24%), while the remaining 4 of the 11 oocytes showed an input conductance decrease (−28.38 ± 5.86%). The increase of the input conductance was correlated with an increased maximal steady-state current, and the decreased input conductance was correlated with a maximal current reduction in every measured oocytes. As mentioned before the existence of two responses was verified with the ANOVA-one way test and showed a very significant value of *p* < 0.001 (Table 3).

**Table 3:** **Quantitative analysis of I/V slope changes at GluN1-4a/b+GluN2A at HP**.

Subunit composition	I/V slope at 0.1 MPa (μS) Mean ± SEM	I/V slope at 5.0 MPa (μS) Mean ± SEM	I/V slope (%Δ) Mean ± SEM	*n*	*p*
GluN1-4a + GluN2A	36.31 ± 6.36	45.31 ± 6.58	42.97 ± 18.24	9	<0.001
	**31.89 ± 6.03**	**24.89 ± 6.21**	**−25.73 ± 8.79**	**5**
GluN1-4b + GluN2A	54.38 ± 7.39	73.36 ± 9.12	37.81 ± 7.24	7	<0.001
	**76.55 ± 16.61**	**54.54 ± 12.69**	**−28.38 ± 5.86**	**4**

*The I/V slope is expressed as mean ± SEM under control (0.1 MPa) and HP conditions (5.1 MPa), mean %**Δ** change ± SEM of I/V; n, number of experiments; p, degree of statistical significance for ANOVA-one way test, which tested the division of the results in two clusters of responses (an increase or a decrease of the current at HP). Both variants GluN1-4a and GluN1-4b exhibited opposing response types, showing an increase (normal font) and a decrease (bold font) of the I/V slope at HP. Each NMDAR subtype was tested with at least two separate batches of oocytes obtained from different frogs, while each batch showed the two types of responses*.

In order to examine the general correlation between changes in NMDARs’ current amplitude and input conductance, the *R*^2^ of the regression line between %Δ in maximal steady-state amplitude and %Δ in I/V slope was recalculated for the two subunits. Because each subunit showed two different responses, a total of four points are shown in the graph of (Figure 2B). The correlation was quite high *R*^2^ = 0.897 and the slope was 0.952. Thus, the “opposing response” is corroborated by the “correct” change in the input conductance. Therefore, the correlation coefficient of the present results is much higher than the previously reported value, obtained for averaged results (Bliznyuk et al., 2015).

### Glutamate Dose Response Analysis

**Figure 3:**
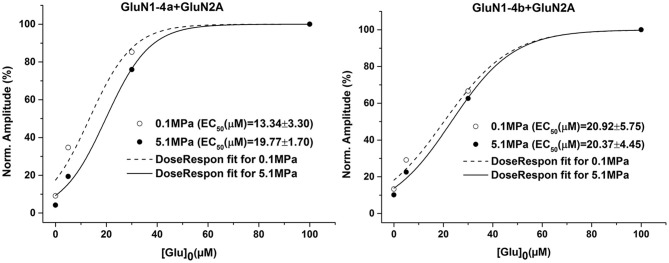
**HP effects on glutamate dose-response.** GluN1-4a/b were co-expressed with GluN2A. There were no significant changes in the dose-response curve and the EC_50_ at HP in both *N*-methyl-D-aspartate receptor (NMDAR) subtypes. The result for each condition were averaged (*n* = 6) and dose-response curve fitting was performed. The GluN1-4b graph has been published earlier in Bliznyuk et al. (2015).

In our previous study (Bliznyuk et al., 2015) we showed that for subunits GluN1-1a/b and GluN1-4b co-expressed with GluN2A there is no statistically significant difference between averaged EC_50_ at control and HP. In order to verify that this is also valid for the GluN1-4a+GluN2A subtype we constructed additional dose response curves (Figure 3) of both increased and decreased current groups. Averaged EC_50_ at control and HP showed no statistically significant difference between increased and decreased response oocytes. This indicates that changes in receptor affinity to the agonists cannot explain neither the pressure effect nor any difference between the two GluN1-4 splice variants.

### Clustering: Mathematical Analysis

**Figure 4:**
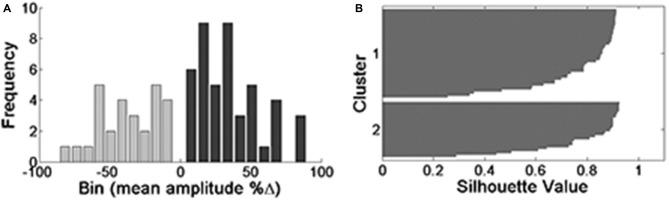
**Clustering analysis of all data (*n* = 73) when *k* = 2.** Histograms are presented on the left **(A)** and the corresponding silhouette values on the right **(B)**. Silhouette values higher than 0.5 are considered significant.

The aforementioned attempts to show at least one possible underlying mechanism for the opposing responses revealed no candidate. However, the experimental results ("New Approach for Analysis" Section) strongly suggest that a real dichotomic phenomenon is involved. In order to strengthen this notion we performed an extended clustering analysis on the largest sample possible, by lumping all the experiments (oocytes) to one batch (*n* = 73 all data, *n* = 44 for positive responses, *n* = 28 for negative responses). Albeit in various groups we used different amounts of injected cRNA, which affected the current amplitudes, since the phenomenon of opposing responses was observed in all of them, the lumping was allowed. Applying a Kolmogorov-Smirnov test revealed that all data together is not distributed normally. Clustering analysis was performed as above (Figure 4), verification of the existence of two different groups of responses was done with the ANOVA-one way test (*p* = 0). Initially, the algorithm performed analysis on an estimated number of two groups (*k* = 2 clusters, Figure 4A). However, both clusters are still not distributed normally.

**Figure 5:**
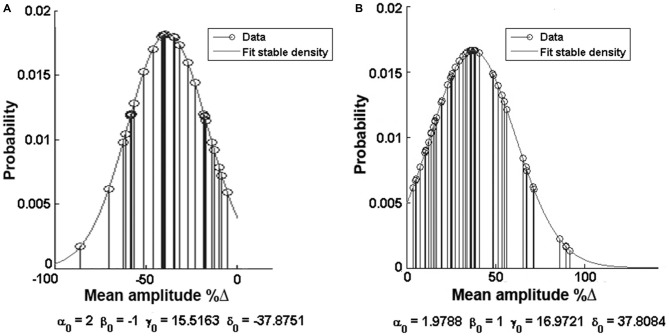
**Alpha function distribution analysis. (A)** Distribution of the negative (decreased) response group. **(B)** Distribution of the positive (increased) response group. Note the values of the Alpha function at the bottom of each histogram.

In order to describe the distribution of the data we used the α-stable distribution function in MatLab^®^, which calculates the distribution values for each of the clusters (Figure 5). The negative response group acquired values of α = 2, β = −1, γ = 15.5163, δ = −37.8751, while the positive response group attained very similar values of α = 1.9788, β = 1, γ = 16.9721, δ = 37.8084.

## Discussion

The mathematical analysis confirmed that the NMDAR subtype that contains GluN1-4a/b splice variants exhibited “dichotomic” responses at HP. In contrast, other subtypes containing GluN2A or GluN2B with different GluN1 variants (GluN1-1a/b, GluN1-2a/b, GluN1-3a/b, see “Introduction” Section) always exhibited current increase.

By using cluster analysis we showed that GluN1-4a/b splice variant-containing NMDARs form two separate groups that responded with either an increase or decrease of the currents under HP conditions. Alpha function distribution analysis showed that the values of the two groups are very similar: α, being responsible for the tail of the distribution, is 1.9788 for the positive group and 2 for the negative group; β, which describes the skewness of the distribution, is 1 and −1, respectively; γ, which describes the scale of the distribution, and δ, being responsible for its location, have very similar values, γ = 16.9721 and δ = 37.8084 for the positive group and γ = 15.5163 and δ = −37.8751 for the negative response. Thus, we may speculate that the mechanism underlying the switch between an increase and decrease of the current at HP is a single process the nature of which is still unknown.

The experimental results however indicated that aggregate formation, type of the GluN2 subunit, and modified stoichiometry are not responsible for the HP “dichotomic” response. Yet, the good correlation between the input conductance changes and the current modifications at HP further support the “single switch” hypothesis.

Recordings were performed in the absence of Mg^2+^ which is a common practice in recombinant NMDARs studies. This may raise the question of the physiological relevance of the findings to CNS physiology. However, we have no reason to suspect that the current behavior and channel open probability will change in the presence of Mg^2+^ at holding potential of *in vivo* −20 to 30 mV which imposes complete Mg^2+^ relief. In fact, it has been demonstrated that even at a membrane potential of −40 mV the I/V curves recorded with or without the presence of the Mg^2+^ are identical (for details see Figure 3; Cavara et al., 2010). Furthermore, we have previously shown (Mor and Grossman, 2010) in rat brain slices that the efficacy of Mg^2+^ block of NMDAR synaptic potential is greatly reduced under HP conditions. This further indicates that the Mg^2+^ effect if any will be negligible.

At present we do not have an explanation for the current increase of NMDAR at HP; we may only speculate about the mechanism. A possible mechanism could be an increase in the amount of the receptors that are exposed on the membrane as a response to the HP due to a modulation in the expression machinery. Alternatively, HP may cause local mechanical alterations that expose more NMDA receptors otherwise inaccessible at the bottom of the microvilli-reach oocytes membrane.

As mentioned in the introduction, increased NMDAR current will cause greater and longer depolarization at the postsynaptic neuron (dendrites) that will cause hyperexcitability: the ability to translate synaptic potentials into higher firing rate (Grossman et al., 2010). This is one of the main features of HPNS. Normally, NMDAR opening is associated with influx of Ca^2+^ (in addition to Na^+^). Small elevation of cytosolic [Ca^2+^] serves as a signal for several processes such as learning and memory (Lee and Silva, 2009). However, under HP conditions, a large increase in NMDAR conductance will allow excessive neuronal Ca^2+^ influx that may be associated with “glutamate toxicity” or even apoptosis (Orrenius et al., 2003). Therefore, it is of great importance to reveal the mechanism by which HP reduces the current in any NMDAR.

The present study strongly supports the idea that the dichotomic response of the GluN1-4a/b splice variants (co-expressed with GluN2A or GluN2B) could be used as a model for studying reduced response in NMDAR at HP. This could provide the key for eliminating the reversible malfunctioning short term effects of HPNS or even the deleterious long term effects induced by increased NMDARs function during HP exposure.

## Author Contributions

AB: study design, acquisition of data, analysis and interpretation of data, drafting of the manuscript. GG: analysis of data, drafting and revision of the manuscript. MH: acquisition of data, analysis and interpretation of data, drafting of the manuscript. YG: study design, acquisition of data, analysis and interpretation of data, drafting of the manuscript.

## Conflict of Interest Statement

The authors declare that the research was conducted in the absence of any commercial or financial relationships that could be construed as a potential conflict of interest.
